# Fluid Biomarkers for Predicting the Prognosis of Liver Cirrhosis

**DOI:** 10.1155/2020/7170457

**Published:** 2020-03-20

**Authors:** Si-Hai Chen, Qin-Si Wan, Ting Wang, Kun-He Zhang

**Affiliations:** Department of Gastroenterology, The First Affiliated Hospital of Nanchang University, Jiangxi Institute of Gastroenterology and Hepatology, Nanchang 330006, China

## Abstract

Liver cirrhosis is the terminal stage of most chronic liver conditions, with a high risk of mortality. Careful evaluation of the prognosis of cirrhotic patients and providing precise management are crucial to reduce the risk of mortality. Although the liver biopsy and hepatic venous pressure gradient (HVPG) can efficiently evaluate the prognosis of cirrhotic patients, their application is limited due to the invasion procedures. Child-Pugh score and the model for end-stage liver disease (MELD) score had been widely used in the assessment of cirrhotic prognosis, but the defects of subjective variable application in Child-Pugh score and unsuitability to all phases of liver cirrhosis in MELD score limit their prognostic values. In recent years, continuous efforts have been made to investigate the prognostic value of body fluid biomarkers for cirrhotic patients, and promising results have been reported. Since the collection of fluid specimens is easy, noninvasive, and repeatable, fluid biomarkers can be ideal indicators to predict the prognosis of cirrhosis. Here, we reviewed noninvasive fluid biomarkers in different prognostic functions, including the prediction of survival and complication development.

## 1. Introduction

Advanced liver cirrhosis (LC) is a life-threatening disorder with limited treatment options [1]. Annually, LC causes 1.03 million deaths worldwide, ranking as the 14^th^ cause of disease death [2]. The patients with decompensated LC present an appropriate 10-fold risk of death compared with the general population [3]. The 6-week mortality of cirrhotic patients with variceal bleeding is about 10%–20% [4]. The mortality of cirrhotic patients with ascites is 14% within 1 year and 44% within 5 years [5]. The development of hepatocellular carcinoma (HCC) may accelerate the death of the disease at any stage. Precise assessment of prognosis followed by efficient therapies, such as endoscopic or pharmaceutical intervention, should be offered to patients with a high risk of death.

Liver biopsy and hepatic venous pressure gradient (HVPG) are able to well reflect the prognosis of LC patients [6–8], but they are limited in clinical applications due to the invasive procedures. Child-Pugh and the model for end-stage liver disease (MELD) scores are currently noninvasive methods to predict the prognosis of LC. However, Child-Pugh score has the defect of subjective variables used. Although the MELD score is more reproducible and accurate than Child-Pugh score in terms of the prediction of prognosis of LC, it is established in candidates for transjugular intrahepatic portosystemic shunt (TIPS); moreover, it is more suitable for end-stage patients [9]. Therefore, simple, reproducible, and noninvasive indicators are required to predict the prognosis of different types of LC patients.

Fluid biomarkers are ideal for predicting LC prognosis since they can be obtained by a simple, noninvasive, and reproducible way. Recently, lots of efforts have been made to explore the prognostic value of fluid biomarkers in different stages of LC. In the present paper, we summarized and discussed these biomarkers based on their own different prognostic value.

## 2. Prognostic Biomarkers for Prognosis of LC

### 2.1. Prognostic Biomarkers of Survival Rate

There were several fluid biomarkers and panels of biomarkers developed to predict the overall survival of cirrhosis.

Red blood cell distribution width (RDW) is a common indicator of routine blood tests and it has a certain prognostic value. Turcato et al. [10] carried out a retrospective study that included 542 patients with acute decompensate LC and showed that the median RDW in this patient cohort was 15.7%; the mortality rate at 1 month was significantly lower in the patients with RDW less than 15.7% than in those with RDW more than 15.7% (8.2% vs. 21.8%). After adjusting for the age, hepatocellular carcinoma, renal function, hemoglobin value, and CLIF-C AD (Chronic Liver Failure Consortium Acute Decompensation) score, the dichotomous and continuous RDW was the independently predictive factor for predicting 1-month mortality (OR: 2.319 and 1.216, respectively). Besides, the model combined with RDW and CLIF-C AD score had a better ability of discrimination than CLIF-C AD (AUROC: 0.769 vs. 0.720; *p*=0.006).

C-reactive protein (CRP) is a nonspecific biomarker of inflammation [11, 12]. Recently, a number of studies showed that CRP had favorable prognostic value in LC. A retrospective study in 336 cirrhotic patients with spontaneous bacterial peritonitis (SBP) showed that the patients with serum levels of CRP ≥5 mg/dL had significantly higher in-hospital mortality (45.5% vs. 28.6%) and lower rate of antibiotic responses (63.5% vs. 83.5%) than those with CRP <5 mg/dL [13]. Another retrospectively observational study enrolled 202 LC patients with sepsis and 87 of them with repeated CRP measurements at day 4 and/or 5; the results showed that the ratio of the follow-up CRP level to the initial CRP level might be a useful prognostic factor to predict the mortality of LC patients (OR: 19.12), but not initial CRP level [14].

Blood leukocyte and neutrophil counts are conventional inflammatory markers. Recently, it is found that neutrophil to lymphocyte ratio (NLR) is a diverse functional prognosis predictor in some diseases, such as myocardial infarction, adult polymyositis and dermatomyositis, and spontaneous intracerebral hemorrhage [15–17]. NLR is related to immune dysregulation in patients with cirrhosis and is inexpensive to measure. Some studies showed that NLR is an emerging prognostic predictor in LC. Kalra et al. [18] evaluated the prognostic value of NLR in liver-related death in low MELD score patients listed for liver transplantation and found that high NLR was associated with liver-related death and independent of MELD score and cirrhosis stage. In another retrospective study, Rice et al. [19] found that increasing NLR was associated with the death of LC patients within 1 year after nonelective hospitalization (HR: 2.17–2.84) and remained significant after adjusting for age, MELD score, hepatocellular carcinoma, and severity of acute-on-chronic liver failure (ACLF).

Aspartate transaminase (AST)/PLT (APRI) is proved to be a simple noninvasive index able to predict fibrosis and cirrhosis in patients with chronic hepatitis C [20]. Additionally, APRI is an independent prognostic index in primary biliary cirrhosis (PBC). Trivedi et al. [21] evaluated 1015 patients with PBC and found that in both derivation and validation cohorts, the patients with higher baseline or one-year APRI values had poorer transplant-free survival.

Serum albumin levels are able to predict the prognosis of LC. In compensated LC patients, serum albumin levels <4 g/dL indicated a worse prognosis at 5 years compared with those with serum albumin levels ≥4 g/dL [22]. In patients with decompensated LC, albumin is changed in both quantity and quality [23]. There are three homodimeric albumin isoforms, hdHA-DA, hdHA-L, and hdHA-native, in patients with cirrhosis and healthy controls. During 1 year follow-up, it was found that patients with a lower relative amount of the hdHA-DA or a higher relative amount of hdHA-native seemed to have a significantly longer survival (10.22 ± 0.44 vs. 8.28 ± 0.56 months; 9.94 ± 0.42 vs. 8.33 ± 0.64 months) [24].

Patients with LC are often complicated with electrolyte disorders. Hyponatremia is a common complication in LC. Several researches had proved that serum sodium levels are well prognostic in LC. Jenq et al. [25] observed 126 patients with LC who admitted to ICU and found that the patients with lower serum sodium concentration (≤135 mmol/L) at the first day of admission had a significantly higher in-hospital mortality (73% vs. 56%) and a poor 6-month survival rate after discharge, and the similar conclusion about 6-month survival was also achieved in the subset of patients with gastrointestinal hemorrhage.

Urokinase plasminogen activator receptor (uPAR) is a membrane glycoprotein. When a body suffers from a tumor, inflammation, and other pathological conditions, uPAR is upregulated and released into the blood circulation by hydrolysis of phospholipase or protease. suPAR is a soluble form of uPAR and a promising prognostic biomarker [26–28]. A study indicated that suPAR could predict the hospital and long-term mortalities in patients with critical illness and sepsis [29]. Zimmermann et al. [30] detected serum suPAR levels in 159 patients with chronic liver disease (98 cirrhosis) and demonstrated that serum suPAR levels were significantly higher in LC patients than those in non-LC patients, and the patients with suPAR levels >9 ng/ml had significantly higher mortality during a median follow-up period of 478 days (RR: 8.5); they also found that suPAR levels were valuable for predicting 28-day mortality of decompensated cirrhosis and independent of MELD score and infection, with hazard ratio 4.83, sensitivity 71%, and specificity 71%, and remained valuable for 90-day mortality (HR: 2.93) [31].

Neutrophil gelatinase-associated lipocalin (NGAL) is a glycoprotein composed of a polypeptide chain with 178 amino acid residues. Due to its small molecular size, it can be filtered to the urine where it can be measured from. It shows diverse biological functions, such as embryonic development, cell differentiation, inflammation and immune response, apoptosis, signal transduction, carbohydrate lipid metabolism, tumor formation, invasion, and metastasis [32]. Recently, some studies reported that NGAL, in both blood and urine, is a novel sensitive biomarker with prognostic value for renal injury in LC patients. Gungor et al. [33] reported that plasma NGAL (pNGAL) had potential ability to predict the outcome of liver cirrhosis in patients with hepatorenal syndrome (HRS): the patients with pNGAL>289.6 *μ*g/L had a shorter survival time during 6-month follow-up, with AUROC 0.819 higher than CTP score (0.795), MELD-Na score (0.807), urine NGAL (0.686), and sensitivity 83.7% and specificity 72.2% in predicting mortality. Ariza et al. [34] investigated serum and urine NGAL in 716 patients in which 148 patients were with ACLF and demonstrated that urinary NGAL (uNGAL) was markedly increased in ACLF compared with non-ACLF patients and was also an independent predictive factor for 28-day transplant-free mortality when combined with MELD score and leukocyte count (AUROC: 0.880).

Patients with LC are often associated with decreased body fat mass and insulin resistance. Resistin, a secreted protein produced by adipocytes and detectable in plasma, plays an important role in insulin resistance [35]. Yagmur et al. [36] detected resistin in 82 patients with chronic liver diseases (including 67 cirrhosis) and followed up for 6 years; they found that resistin levels were significantly elevated in cirrhosis patients compared with healthy controls and positively correlated with the stage of cirrhosis. However, da Silva et al. [37] did not observe a significant relationship between resistin levels and transplant-free survival: they detected plasma resistin and adiponectin levels and found that adiponectin, but not resistin, levels were associated with lower survival (HR: 1.034).

Copeptin is a fragment derived from the vasopressin precursor. Vasopressin is increased and plays a role in the development of complications in patients with cirrhosis. The measurement of vasopressin is difficult, but copeptin is more stable and easier to be detected in the blood. Moreno et al. [38] reported that plasma copeptin levels were increased along with the severity of liver disease in cirrhotic patients, with a 2.3-fold risk of death/liver transplantation in high-level patients compared with low-level ones, and independently predicted the 1-year mortality or liver transplantation (AUROC: 0.7). Kerbert et al. [39] investigated 61 LC patients at the waiting list for liver transplantation and found that patients with low levels of serum copeptin had a significantly higher transplant-free survival rate than those with high levels of copeptin. Subsequently, in 184 hospitalized cirrhotic patients, they demonstrated that patients with lower copeptin levels had a better transplant-free survival rate during 6 and 12 months of follow-up [40].

M30, M65, and M65EpiDeath belong to cytokeratin 18- (CK-18-) based serum markers reflecting apoptotic or overall epithelial cell death. It was found that the increased serum levels of a fragment of CK-18 were associated with liver fibrosis and acute or chronic liver diseases [41–43]. Sekiguchi et al. [44] detected M30, M65, and M65EpiDeath in 130 PBC patients and found that they were all significantly higher in PBC than in healthy controls and were significantly correlated with fibrosis stage; the mortality was significantly higher in patients with high M65EpiDeath levels (HR: 6.13). Waidmann et al. [45] prospectively investigated 211 LC patients and found that these three CK-18-based cell death markers also could be potential biomarkers of severity of LC induced by other etiologies (mainly alcohol abuse and HCV infection), but only M65EpiDeath was associated with the patients prognosis, and interestingly, M65EpiDeath level was a factor for mortality in patients with compensated LC (HR: 11.483), but not decompensated LC.

Insulin-like growth factor-binding protein (IGFBP) is a group of polypeptides that are homologous with insulin. IGFBP-3, the most abundant type of IGFBPs in blood circulation, is mainly synthesized in Kupffer cells of the liver [46]. It is an indicator that can reflect the liver capacity. A study showed that serum IGFBP-3 was lower in cirrhotic patients than healthy controls, which indicated that low IGFBP-3 was associated with hepatocellular dysfunction [47]. Correa et al. [48] found that lower IGFBP-3 levels were associated with worse outcomes in patients with cirrhosis: the low IGFBP-3 group had lower survival rates than high IGFBP-3 group in outpatients followed up for a median of 20 months (72.1% vs. 88.6%) and in hospitalized patients at 90-day transplant-free survival (56.1% vs. 80.4%).

Pentraxin-3 (PTX-3) is a 40 kDa protein that contained 381 amino acids. Many cells are able to produce PTX-3 under inflammatory stimulation [49]. Narciso-Schiavon et al. [50] analyzed the circulating PTX3 levels in cirrhotic patients and found that higher PTX-3 levels indicated a significantly lower 90-day survival rate (54% vs. 77%). Fan et al. [51] also found that acute decompensated patients with high PTX-3 levels had a higher probability of in-hospital mortality (HR: 3.94) and 3-month mortality (HR: 3.52).

Myostatin is a cytokine able to strongly suppress skeletal muscle growth [52]. The serum concentration of myostatin in LC patients was significantly higher than the healthy [52, 53]. Nishikawa et al. [54] measured serum myostatin levels in 198 LC patients, and the results showed that the overall survival rates of patients with high myostatin were significantly lower than those with low myostatin: 1-, 3-, 5-, and 7-year cumulative survival rates were 96.0%, 77.9%, 53.0%, and 39.1%, respectively, in the high myostatin group and 96.4%, 87.6%, 77.6%, and 73.2%, respectively, in the low myostatin group after excluding hepatocellular carcinoma patients at baseline.

The levels of Wisteria floribunda agglutinin^+^-colony-stimulating factor 1 receptor (WFA^+^-CSF1R) could reflect the progression of liver fibrosis and possibly evaluate the severity of LC [55]. Lio et al. [56] detected the levels of WFA^+^-CSF1R and total-CSF1R in 214 patients with liver diseases caused by HCV infection and found that patients with LC (*n* = 115) had significantly higher plasma levels of WFA^+^-CSF1R and total-CSF1R than the patients (*n* = 99) with chronic hepatitis (208.9 ng/ml vs. 82.3 ng/ml; 845.3 ng/ml vs. 536.4 ng/ml, respectively) and that the mortality was significantly higher in patients with WFA^+^-CSF1R levels ≥310 ng/ml (HR: 1.8), with an AUROC of 0.691.

Wisteria floribunda agglutinin positive Mac-2 binding protein (WFA^+^-M2BP) is a novel and noninvasive biomarker for the assessment of liver fibrosis [57, 58]. Yamasaki et al. [59] enrolled 707 hepatitis C patients including 120 cases of LC and followed up for 20 years, and the results showed that the patients with the serum levels of WFA^+^-M2BP 1-4 and ≥4 at admission presented higher risk to develop HCC compared with WFA^+^-M2BP <1 (HR = 5.115 and 8.318, respectively). In PBC patients, ones with serum WFA^+^-M2BP levels <2.0 had a significantly higher survival rate of 10 years compared with the patients whose WFA^+^-M2BP value ≥2.0 (98.8% vs. 59.1%) [60].

Interleukin-22 (IL-22) is a crucial parameter of pathology in experimental liver damage and associated with the prognosis of LC. Kronenberger et al. [61] detected the systemic levels of IL-22 in 120 LC patients and 40 healthy donors and found that IL-22 was more detectable in LC patients compared with healthy donors (74.1% vs. 10%), and the patients with elevated IL-22 levels had a reduced survival time (321 days vs. 526 days).

Lysophosphatidic acid (LPA), a secreted glycoprotein formed from lysophosphatidylcholine by autotaxin (ATX) which possesses both phosphodiesterase and lysophospholipase D activity, activates hepatic stellate cells and inhibits their apoptosis. A prospective cohort study included 270 cirrhotic patients and found that low levels of serum autotaxin indicated a longer survival time (HR: 0.575) [62].

Vitamin D is a necessary fat-soluble vitamin in the body, and its deficiency is associated with various chronic liver diseases [63]. A study prospectively recruited 324 patients with alcoholic liver disease in which there were 254 LC patients and were followed up for one year, and the low levels of vitamin D were associated with low overall survival time (HR: 5.95) [64].

Several biomarkers detected in urine also could predict the survival rate of patients with LC. Interleukin-18 (IL-18) is a proinflammatory protein able to regulate immune responses. Urinary IL-18 is associated with AKI and 90-day mortality in critically ill patients [65]. Recently, Tsai et al. [66] detected the concentration of the urinary IL-18 in 168 consecutive cirrhotic patients with severe sepsis and found that it was significantly higher in hospital survivors than in nonsurvivors and was an independent factor to predict mortality of the patients in the hospital. Monocyte chemoattractant protein-1 (MCP-1) is one of the key chemokines in regulating migration and infiltration of monocytes/macrophages and involved in various diseases. Graupera et al. [67] prospectively collected 218 patients with acute decompensated cirrhosis with a follow-up for at least 3 months and detected the levels of MCP-1, osteopontin (OPN), and trefoil-factor 3; the results showed that the levels of urinary MCP-1 were associated with 3-month readmission and death in univariate analysis, and in multivariate analysis, MCP-1 was an independent biomarker to predict 3-month hospital readmission (HR: 2.1) and the event combined with readmission or death (HR: 2.4).

Saliva samples can be collected easily, repetitively, and noninvasively. It is found that the saliva contains biomarkers associated with LC prognosis. Salivary caffeine clearance (CCl) could be used to evaluate the liver function and liver metabolic capacity [68, 69]. Jover et al. [70] calculated the CCl in 34 cirrhotic patients and followed up those patients for average 33.8 months and found that the survivors had a significantly higher CCl than the nonsurvivors (0.43 vs. 0.14) and CCl was an independent factor for predicting survival. However, the value of CCl is susceptible to other factors, such as treating with drugs that alter the hepatic metabolism of caffeine; thus, the application of CCl might be restricted in the clinic. Dysbiosis or altered gut microbiota in cirrhotic patients' stool and colonic mucosa would be likely associated with disease severity and systemic inflammation [71, 72]. Interestingly, salivary dysbiosis is related to the prognosis of LC patients. Bajaj et al. [73] enrolled 102 cirrhotic patients and found that the patients who were hospitalized within 90 days had a lower salivary dysbiosis ratio compared with the patients' free hospitalization (0.15 ± 0.24 vs. 0.52 ± 1.2) and salivary dysbiosis ratio was an independent predictor of 90-day hospitalization (OR: 0.5).

### 2.2. Prognostic Biomarkers of Complication of Cirrhosis

#### 2.2.1. Predicting the Renal Injury

It is not uncommon that cirrhotic patients combine with acute renal injury (AKI) that is associated with mortality [74]. Cirrhotic patients with renal failure had over 7-fold risk of death within 1 year compared with patients without renal failure [75]. Therefore, it is necessary to diagnose kidney injury early and implement effective strategies timely. Actually, serum creatinine (Cr) and its derived formulae in estimating glomerular filtration rate (GFR) are inaccurate to assess renal function [76].

Cystatin C (CysC) has been proposed as a specific marker for detecting a reduced GFR and an early indicator of impaired renal function. It is superior to Cr because its expression level is independent of age, sex, muscle mass, and inflammatory or malignant diseases [77]. HRS is a functional renal failure caused by intrarenal vasoconstriction [78]. Sharawey et al. [79] evaluated 80 cirrhotic patients with ascites and normal serum creatinine levels (<1.2 mg/dL) and found that serum CysC was a predictor of HRS development (OR: 2.1); when the cut-off value of serum CysC was set at 1.819 mg/L, the area under receiver operating characteristics curve (AUROC) for predicting the development of HRS within 12 months was 0.86, and the corresponding sensitivity and specificity were 88.8% and 67.7%, respectively. CysC was also a predictor for predicting the development of ACLF. Recently, Markwardt et al. [80] measured the plasma levels of CysC in 429 patients hospitalized for acutely decompensated cirrhosis and results showed that the baseline CysC level was not only a predictor of renal dysfunction (OR: 9.4) but also a predictor of HRS, ACLF, and 90-day mortality (OR: 4.2, 5.9, and 3.1, respectively). Maiwall et al. [81] developed AKI-Score including bilirubin, Cystatin C, and prior AKI to predict the new AKI in patients with LC. The C-index of the AKI-Score was 0.78 and 0.80 in training and validation groups, respectively, which was higher than MELD (0.71 and 0.73 in training and validation groups, respectively) and CTP (0.69 and 0.75 in training and validation groups, respectively). Besides, the AUROC of the AKI-Score (0.81 and 0.85 in training and validation groups, respectively) also was higher than the MELD score (0.70 and 0.74 in training and validation groups, respectively) and CTP (0.67 and 0.76 in training and validation groups, respectively).

NGAL is expressed in a variety of normal human tissues, such as bronchial epithelial cells, gastric wall cells, and proximal renal tubular epithelial cells. Nevertheless, NGAL shows a heterogenous expression in the kidney, positive in proximal renal tubule epithelial cells, while negative in the distal renal tubule, glomerulus, and renal units [82]. Barreto et al. [83] found that levels of uNGAL were markedly higher in patients with AKI compared with ones without, with predictive value for persistent AKI (OR: 1.48) and 3-month survival rate (HR = 1.10); interestingly, patients with higher uNGAL levels had a more probability to develop a second infection during hospitalization. Verna et al. [84] measured the levels of uNGAL in 118 patients with cirrhosis and also found that uNGAL could be a potential biomarker for predicting the mortality of inpatient with cirrhosis (OR: 6.05).

Aquaporin-2 (AQP2) is a water channel sensitive to vasopressin and located on the luminal side of collecting ducts of the kidney [85]. A portion of AQP2 can be excreted into urine and be detected. Busk et al. [86] analyzed baseline concentration of urine AQP2 in cirrhotic patients, and the results showed that it was able to significantly distinguish patients with the progression of renal insufficiency from the patients without and could be a predictor for 28-day mortality but not for 90-day mortality.

Kidney injury molecule-1 (KIM-1) is a type l transmembrane glycoprotein with a potential for the detection of tubular injury in renal diseases. Yap et al. [87] prospectively observed cirrhotic patients with normal serum creatinine and Child grade B or C for 12 weeks and found that urinary KIM-1 levels could be a potential biomarker for predicting the development of HRS in progressive cirrhotic patients (RR: 1.973), with AUROC of 0.78.

#### 2.2.2. Predicting the Infection

LC patients are frequently complicated with a bacterial infection. A bacterial infection could promote the process of cirrhosis and eventually result in patient death [88, 89]. Preventive effective measures of cirrhotic patients with a high risk of infection might potentially decrease mortality. Several biomarkers had been found to have a potential ability to predict the infection in LC.

Lipopolysaccharide-binding protein (LBP), an acute-phase protein produced by hepatocytes, is found to be a biomarker able to predict the development of severe infection in LC patients. High serum LBP levels in the cirrhotic patients with ascites developing severe bacterial infection were significantly higher than those in patients with normal LBP levels (32.4% vs. 8.0%; RR: 4.49) [90]. LBP is a predictor of LC prognosis. The authors of [91] detected LBP levels in 88 patients with decompensated cirrhosis including 18 patients with infection and the others without infection and found that, in noninfective patients, those with lower level of LBP had a lower 90-day mortality than the high level (48.0% vs. 24.4%), and only high LBP (HR: 8.1) and MELD (HR: 1.1) were predictors of mortality in multivariate analysis, but this association was not observed in infective patients. In 58 critically ill cirrhotic patients with severe sepsis, Chen et al. [92] found that the cumulative survival rate at 28 days was higher in the high serum LBP group (>46 ng/mL) than that in the low serum LBP group (<46 ng/mL) (72.7% vs. 16.7%), with the AUROC of 0.809, sensitivity of 72.7%, and specificity of 83.3%. In addition, Papp et al. [93] did not observe an association between LBP levels and the probability of developing infections during a 3-month follow-up period in LC patients. Therefore, the value of LBP in the evaluation of infection and prognosis in LC patients needs more studies.

High blood levels of free cortisol indicate the severity of cirrhosis. Thevenot et al. [94] prospectively detected serum free cortisol (SFC) in 95 hemodynamically stable cirrhotic patients and found that the patients with levels of SFC ≥79 nmol/L had a significantly higher one-year mortality (26.2% vs. 3.4%). Another study including 143 LC patients with acute decompensation showed that patients with relative adrenal insufficiency had a higher probability of infection (41% vs. 21%), sepsis (27% vs. 9%), and death (22% vs. 7%) during follow-up of 3 months [95].

#### 2.2.3. Predicting Gastroesophageal Varices

Acute variceal bleeding is a serious complication of LC. Despite the progress in the standardization of the supportive care and new therapeutic approach, the 6-week mortality of AVB was around 20% [96]. In the clinic, lacking fluid biomarkers could predict the occurrence of gastroesophageal varices and AVB.

Theoretically, lower platelet (PLT) counts indicate a higher risk of bleeding and poor prognosis in LC patients, but it is not supported by reports. Qamar et al. [97] found that, in patients with LC and portal hypertension, PLT counts could not predict the presence of gastroesophageal varices at admission and during follow-up. Other studies showed that PLT number was not a risk factor of major bleeding during a four-year observation period [98] and not a predictor of survival rate in patients with LC [99]. Kim et al. [100] found that the index named P2/MS, (PLT)^2^/[monocyte fraction (%) × segmented neutrophil fraction (%)], was negatively associated with the presence of esophageal varices in hepatitis B viral cirrhosis, and the patients with P2/MS ≥25 had lower risk of bleeding esophageal varices [101].

Patients with LC are often complicated with a disorder nutritional status, like protein-energy malnutrition (PEM) and sarcopenia, which is associated with the prognosis of LC [102]. Branched-chain amino acids (BCAAs) are energy substrates in muscles. Studies proved that cirrhotic patients had lower levels of BCAAs, but higher levels of aromatic amino acids (AAAs) [103, 104]. Ishikawa et al. [105] calculated the BCAA-to-tyrosine ratio (BTR) in 530 cirrhotic patients and found that patients with BTR <4 have more events including the worsening of esophageal and/or gastric varices, death, HCC, and liver failure (total events 53.3% vs. 11.8%, HR: 6.34).

### 2.3. Prognostic Biomarkers of Cirrhosis with ACLF

Acute-on-chronic liver failure (ACLF), characterized by multiorgan failure, is the major cause of death in patients with acute decompensation of LC, and the mortality rate of patients with ACLF at 90 days is around 50% [106]. It is necessary to seek indicators for early diagnosis of ACLF.

Piano et al. [107] followed up 466 outpatients with cirrhosis and found that the baseline hemoglobin (HR = 0.07) is an independent prognostic biomarker of predicting the development of ACLF at one year.

uNGAL is a multifunctional biomarker to predict the prognosis of LC. Ariza et al. [34] found that uNGAL was not only a biomarker that is independently associated with the ACLF (OR: 1.34) but also could predict the development of ACLF. After adjusting for the INR, Cr, and leukocyte count, the uNGAL still was an independent prognostic factor of the development of ACLF during hospitalization (OR: 1.31).

As previously described, the elevated copeptin levels indicated the higher short-term mortality, and the baseline serum copeptin was also a predictive biomarker of ACLF development. Kerbert et al. [108] followed up the patients without ACLF and identified that the copeptin was the independent prognostic factor for predicting the development of ACLF (OR:1.40).

### 2.4. Prognostic Biomarkers of Cirrhosis with Cardiovascular Dysfunction

Cirrhosis is associated with cardiovascular dysfunction. Several studies had proved that patients with cirrhosis were complicated with impaired cardiac contractility and performance [109, 110]. Brain natriuretic peptide (BNP) and its precursor NT-proBNP and C-type natriuretic peptide (CNP) are traditional biomarkers for evaluating cardiac dysfunction. Actually, those biomarkers had prognostic value in patients with LC. An earlier study found that increased BNP and NT-proBNP were associated with the severity of liver disease [111]. Pimenta et al. [112] found that BNP was an independent predictor of medium-term survival in decompensated cirrhosis. Higher serum BNP levels at preoperative liver transplantation or first postoperative day indicated a higher mortality rate [113, 114]. NT-proCNP is also associated with adverse prognosis of cirrhosis. Koch et al. [41] found that serum NT-proCNP was elevated in advanced liver diseases and indicated unfavorable prognosis when NT-proCNP levels >2 pmol/L (sensitivity 66.7%, specificity 72.8%, RR: 5.4).

### 2.5. Fluid Biomarkers to Improve the MELD Score and CTP

CTP and MELD are the most common score system to evaluate the prognosis of LC and have been widely used in clinical practice. Some studies attempted to incorporate prognostic biomarkers into CTP and MELD scores for improving the performance of the prediction of the prognosis of LC.

MELD-Na score, combined with serum sodium and MELD score, was a presentative improvement of the MELD score. Heuman et al. [115] demonstrated that, in patients with a low MELD score (<21), the MELD-NA score had a better performance than the MELD score in predicting the 180-day mortality (c-statistic: MELD-Na vs. MELD: 0.768 vs. 0.638).

Combination of CRP and procalcitonin (PCT) with MELD score could improve the prediction of 30-day mortality (c-statistic: MELD-CRP, MELD-PCT, MELD-CRP-PCT vs. MELD: 0.79, 0.80, 0.81vs. 0.76) and 90-day mortality (c-statistic: MELD-CRP, MELD-PCT, MELD-CRP-PCT vs. MELD: 0.83, 0.84,0. 85vs. 0.81), and the similar results were obtained from the Mayo Clinic external validation cohort in the prediction of 30-day mortality (c-statistic: MELD-CRP vs. MELD:0.71 vs. 0.67) and prediction of 60-day mortality (c-statistic: MELD-CRP vs. MELD:0.69 vs. 0.65).

Kim et al. [116] established a model that combined serum sodium concentration with MELD score and found the MELD-Na score might have better performance in the assignment of priority of transplantation than MELD.

MELD-Cystatin Score showed a better performance than the MELD score and CTP score in predicting the probability of survival during a year follow-up (C-index: 0.84 vs. 0.83 and 0.74) [81]. But, Finkenstedt et al. [117] established a CysC-based MELD score (replace creatinine with CysC) and it was not superior to MELD score in predicting the 90-day mortality among LC patients. More studies are needed to clarify the prognostic value of incorporating CysC in the MELD model.

As mentioned, uNGAL is a biomarker that could predict the survival and occurrence of complications in patients with LC and also enhance the prognostic value of the MELD score. A model combined with uNGAL and MELD had a better performance in predicting the 28-day transplant-free mortality of LC patients (AUROC: model Vs. MELD: 0.86 vs. 0.81, *p*=0.017) [34].

## 3. Conclusions

Liver biopsy and the measurement of HVPG can efficiently predict the prognosis of LC, but the invasive feature limits their applications in clinical practice. Although Child-Pugh score and MELD score are widely applied to evaluate the prognosis of LC, their drawbacks are obvious, such as subjectivity and incomprehensive scope of application. Accumulating evidences have shown that fluid biomarkers provide prognostic information of liver cirrhosis. Fluid biomarkers are objective measures and relative to different prognostic aspects of cirrhotic patients, including short- and long-term survival, complication development, and disease progression, and some biomarkers are “multifunctional”; for example, CysC predicts not only the mortality of LC but also the development of HRS. Importantly, fluid biomarkers have been found in blood, urine, and saliva, and all these fluids can be collected easily, repetitively, and noninvasively, which is favorable for clinical application. Some studies also attempted to incorporate those prognostic biomarkers into Child-Pugh and MELD score and got a better performance.

However, before these fluid biomarkers are applied to evaluate LC prognosis in clinical practice, several challenges are needed to be overcome, including clinical value to be confirmed by more studies, standardization of detection methods, and comparison with current methods in large samples. The overcoming of these obstacles will facilitate the utilization of fluid biomarkers in the prediction of LC prognosis and promote the precise management of LC patients by a combination of different biomarkers.
